# Microbial immigration across the Mediterranean via airborne dust

**DOI:** 10.1038/srep16306

**Published:** 2015-11-06

**Authors:** Riccardo Rosselli, Maura Fiamma, Massimo Deligios, Gabriella Pintus, Grazia Pellizzaro, Annalisa Canu, Pierpaolo Duce, Andrea Squartini, Rosella Muresu, Pietro Cappuccinelli

**Affiliations:** 1 Department of Biology, University of Padova, Via Ugo Bassi 58/b, 35131 Padova, Italy; 2 Department of Biomedical Sciences-University of Sassari, Italy; 3 Institute of Biometeorology-National Research Council (IBIMET-CNR), Italy; 4 Institute of Animal Production Systems in Mediterranean Environments-National Research Council (ISPAAM-CNR), Italy; 5 Department of Agronomy Animals, Food, Natural Resources and Environment, DAFNAE, University of Padova, Viale dell’Università 16, 35020 Legnaro (Padova) Italy

## Abstract

Dust particles lifting and discharge from Africa to Europe is a recurring phenomenon linked to air circulation conditions. The possibility that microorganisms are conveyed across distances entails important consequences in terms of biosafety and pathogens spread. Using culture independent DNA-based analyses via next generation sequencing of the 16 S genes from the airborne metagenome, the atmospheric microbial community was characterized and the hypothesis was tested that shifts in species diversity could be recorded in relation to dust discharge. As sampling ground the island of Sardinia was chosen, being an ideal cornerstone within the Mediterranean and a crossroad of wind circulation amidst Europe and Africa. Samples were collected in two opposite coastal sites and in two different weather conditions comparing dust-conveying winds from Africa with a control situation with winds from Europe. A major conserved core microbiome was evidenced but increases in species richness and presence of specific taxa were nevertheless observed in relation to each wind regime. Taxa which can feature strains with clinical implications were also detected. The approach is reported as a recommended model monitoring procedure for early warning alerts in frameworks of biosafety against natural spread of clinical microbiota across countries as well as to prevent bacteriological warfare.

Global transport of desert dust is believed to play an important role in many geochemical, climatological, and environmental processes^1^,^2^,^3^,^4^. Dust can convey minerals and nutrients^5^, but also pollutants^6^ and viable microorganisms of various species^7^,^8^, some of which are capable of harming human, animal, plant, and ecosystem health^9^,^10^,^11^. Given the complex and extended routes of meteorological circulation, airborne dust can transport microorganisms for long distance also across sea and mountain range barriers. Benefits of a deeper awareness of potentially inflowing bacteria, fungi and viruses are envisaged in the fields of human healthcare, veterinary prophylaxis, agricultural crop protection and warfare monitoring. Ground-level technologies have strived to guarantee efficient protection against airborne infections arising in fully air-conditioned buildings, in particular preventive measures for hospitals, and their effectiveness have been long since primary concerns^12^. Airborne dust contains bacteria, viruses, fungi and their spores but the number of culturable microorganisms in this life-limiting habitat is rather low, scoring values well below 1% of the live cells^13^. Culture-based studies suggest that the microbial densities can vary across seasons and environmental conditions^14^. To complement the inefficiency of the culture-dependent census^15^, direct molecular approaches within the metagenetics framework have been undertaken to unravel the community composition of prokaryotes and eukaryotes.

The emergence of new infectious diseases in many parts of the world^16^ and the resurgence of old ones like tuberculosis and meningitis^17^,^18^, evidenced the global-threat aspect of the consequences related to airborne transportation of microbes across nations and continents.

The atmosphere-dispersed particulate matter, constituted by a mixture of particles, containing variable fractions of natural solids and pollutants^19^,^20^,^21^, represents a carrier able to passively convey an array of different microorganisms. As a consequence, emission of particle-associated bacteria, fungi and viruses determined by the transcontinental displacement via meteoric circulation (water particles and ensuing rainfall), constitutes i) an effective means of spreading for pathogens and ii) a continuous process of expansion of their biogeographical ranges.

After 2000, the U.S. Geological Survey in St. Petersburg, FL, began the USGS Global Dust Program (initially funded by NASA) to investigate whether live microorganisms would be consistently transported in dust masses (initially in Saharan dust^22^). Authors at USGS using DNA sequencing of the ribosomal gene, were able to isolate and identify over 200 viable bacteria and fungi in samples from St. John in the U.S. Virgin Islands in 2000 during dust air-driven particle transport events^23^.

Many of the viable microorganisms identified in Saharan dust are known from clinical records to cause respiratory diseases (allergic reactions, asthma, and pulmonary infections), cardiovascular diseases, or skin infections^24^. Other microbes in airborne dust are known to be pathogenic to humans, including those causing plague, anthrax, tuberculosis^23^,^26^, or towards livestock, relatively to foot and mouth disease^10^ or to plants, causing sugarcane rust, and other pathologies of cotton, peach, rice and beans^27^,^28^,^29^.

Moreover, the association between microorganisms and air pollutants like PM_2.5_ and PM_10_ (particulate matter with diameters less than 2.5 and 10 μm, respectively)^30^ could represent a recently evolved transfer-mechanism supporting and increasing their natural dust-mediated dispersion and opening new ways for their epidemic diffusion.

Facing the African Continent, Europe is subjected to a large-scale dust-transportation. It has been estimated that 80–120 Tg of dust per year are carried across the Mediterranean towards Europe^31^,^32^. In particular, dust transported by winds can reach an elevation up to 8 km in the atmosphere over the Mediterranean basin^33^.

Every dust-carrying event can be very different from others of the same kind; in fact dust transport and concentration in the air can vary remarkably depending on the synoptic situation^34^. In general the main meteorological scenarios that originate the transport of dust towards the central and western Mediterranean Basin are characterized by the presence of four different synoptic conditions: i) a strong North African thermal low pumps dust till the mid-troposphere, where the western side of a high pressure centered slightly westward transports the plume; ii) the eastern side of an Atlantic trough with the western side of the associated ridge, located between Tunisia and Libya is able to originate an all-level transport; iii) the western side of a well-structured high on the central Mediterranean Basin can supply the flow for low level transport; iv) the north-eastern side of a sea level low centred in south Libya can create the condition for a low level transport^35^,^36^.

Its geographic position and seasonal weather conditions make Italy one of the first frontiers subjected to a natural dust-transportation. Satellite data and forecasting models foretell geographical dispersion of African dust, revealing that the main sources are the Sahara and Sahel regions, located in the north-western part of Africa. Dust events that reach Italy are more frequent in the May-November period, but can also take place in the December-April period^37^.

In the western side of insular Italy, surrounded by the Mediterranean sea, the Sardinia island is located at 130 Nm (240 km) from the North-African coastline and represents a cornerstone for the above phenomena as well as an ideal observation point for monitoring of dust transportation. Dust outbreaks in Sardinia were already described^38^,^39^,^40^ but there is a lack of studies on the microbiological diversity of dust-loaded winds discharging particles from Africa.

The present study addressed the bacterial community of airborne particles in a culture-independent analysis of the 16 S-rRNA genes by high-throughput sequencing. In parallel culturable biota was also analyzed in order to characterize which species remain viable after long-distance atmospheric transport. Two sampling sites were considered, Cagliari and Sassari, covering the whole south-to-north axis aligned with the African winds direction. Two distinct meteorological conditions were chosen; i) a dust outbreak conveying dispersed particles of Saharan origin ii) a baseline situation (negative control) represented by air masses of European origin incoming from the north-western quadrant.

## Results

### Choice of dust-carrying and control conditions

**Figure 1:**
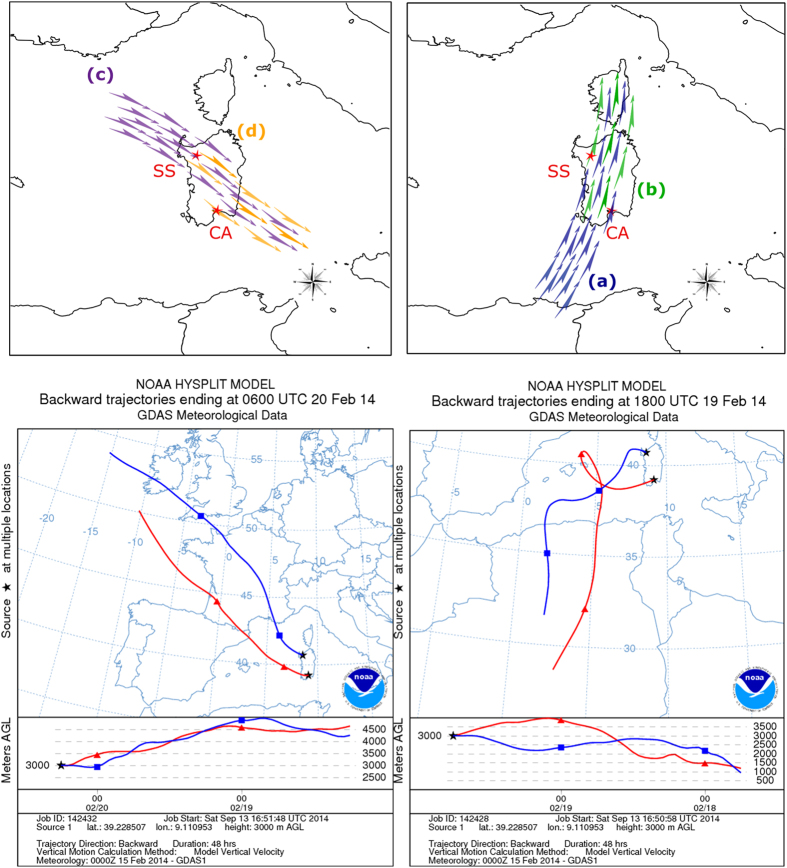
Reconstruction of the wind flows crossing the two sites during the sampling dates. Top left panel: control conditions event with winds blowing from northwest to south-east, Top right panel: African dust-carrying event with northbound winds. Bottom panels: corresponding particle back-trajectories simulations evaluated by the HYSPLIT model for the two sampling days. SS: Sassari sampling point; CA: Cagliari sampling point. Letters and colored arrows in the two top panels indicate four theoretically distinct pools of putatively airborne bacteria: (**a**) from Africa and Mediterranean sea (blue arrows); (**b**) the same plus those raised from inland Sardinia in northbound wind motion from Cagliari to Sassari (green arrows); (**c**) from continental Europe and Tyrrhenian sea (violet arrows); (**d**) the same plus those raised from inland Sardinia in southbound wind motion from Sassari to Cagliari (orange arrows). Source of images: top panels: this work; bottom panels: output of the public website service software HYSPLIT (http://ready.arl.noaa.gov/HYSPLIT.php).

Scheme and conditions are shown in Fig. 1. The sampling was done by considering four-days time laps. Back trajectories plots confirmed the intrusion of African air masses occurring on February 15^th^ through 19^th^ 2014. On the subsequent period from February 20^th^, the above circulation regime halted, high pressure cells over Europe caused opposite winds blowing from northeastern origin, which passed over Italy and Sardinia and allowed to collect samples representing the dust-free control conditions.

The first intrusion of African air masses occurred on the 15th-17th, mainly affecting the PM_10_ levels in the north of Sardinia. On the 18th and 19th February 2014 air masses loaded with dust crossed the Mediterranean northward and eastward towards Italy and Sardinia, affecting the whole island. The analysis of the PM_10_ daily pattern registered in February at the sampling sites showed increasing values during the dust event at both sites with a marked decrease on the 20th (Supplementary Figure S1). The arrival of air masses loaded with dust from North Africa was confirmed by satellite imagery (Supplementary Figure S2).

### Plate-culturable biota

As regards the results of the culture-dependent approach, Fungi were more abundantly isolated and mostly represented by *Aspergillus* spp., while among bacteria *Bacillus* spp. was the leading culturable taxon. Species identified included *Bacillus simplex, Paenibacillus amylolyticus, Brevibacillus formosus, Bacillus megaterium, Bacillus cereus, Kocuria rosea*.

### Culture-independent 16S sequencing analysis

The mean quantitative results of the NGS sequencing in the different samples are shown in Table 1.

The qualitative results of the sequencing annotation allowed the identification of several hundreds of operational taxonomical units per sample. The data at genus rank level are shown in Table 2 where the number of genera and the corresponding ecological indexes calculated from the community data are reported. The full array of taxa can be browsed as spreadsheet in the supplementary material (Supplementary Table S1).

**Figure 2:**
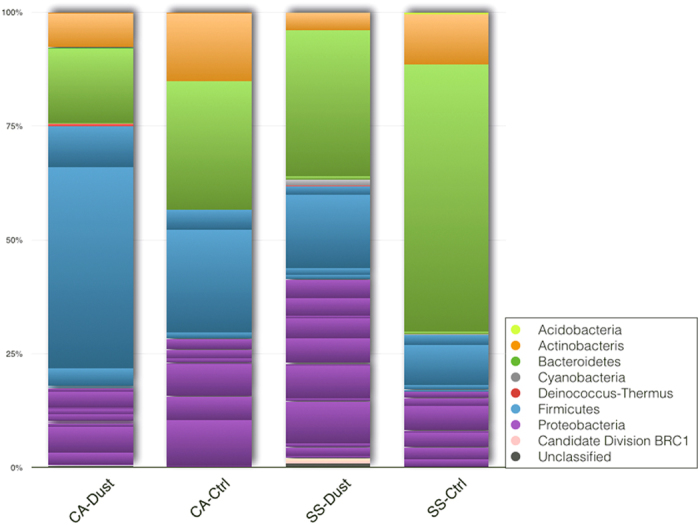
Percentages of sequences assignable to database-identifiable bacterial in each of the two sampling sites at each event (Control or Dust). CA: Cagliari sampling site, SS: Sassari sampling site. The slices within each phylum block of the same color indicate the number of orders found within that phylum.

The most abundant phyla were in all samples Proteobacteria, Firmicutes, Bacteroidetes and Actinobacteria as shown in Fig. 2. A higher amount of orders belonging to Proteobacteria can be observed in the SS-Dust community. Higher percentages of Firmicutes (the Bacillales order in particular), characterize the two Cagliari samples (CA-Dust and CA-Ctrl).

**Figure 3:**
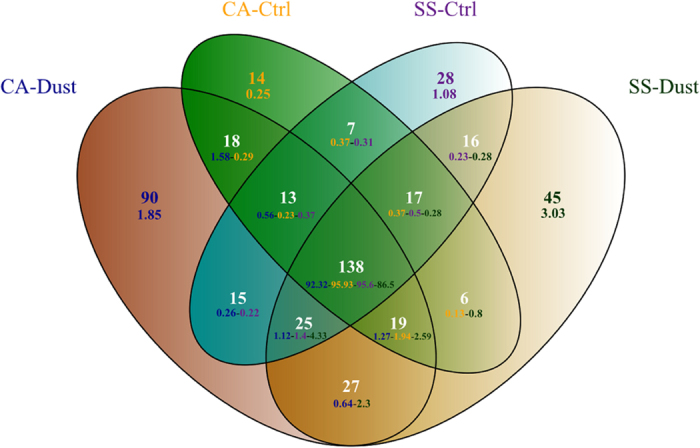
Venn diagram showing the number of specific or shared genera identified in each of the four locations/conditions and the relative percentages (smaller font digits).

The prevailing occurrence of a common core of shared taxa is shown in detail by the overlapping Venn diagrams representation in Fig. 3. The highest proportions of taxa were those shared by all four, 92.3% and 95.9%, 85.6% and 95.6% for Cagliari-Dust, Cagliari-Control, Sassari-Dust and Sassari-Control, respectively, consisting in 138 genera. Conversely, the non-overlapping occurrences were all within frequencies ranging from 0.25% (Cagliari Control) and 3.03% (Sassari Dust). Although limited in comparison to the constant core higher numbers of event-specific genera occurred for the two dust-exposed samplings, with 90 and 45 for Cagliari and Sassari, respectively.

**Figure 4:**
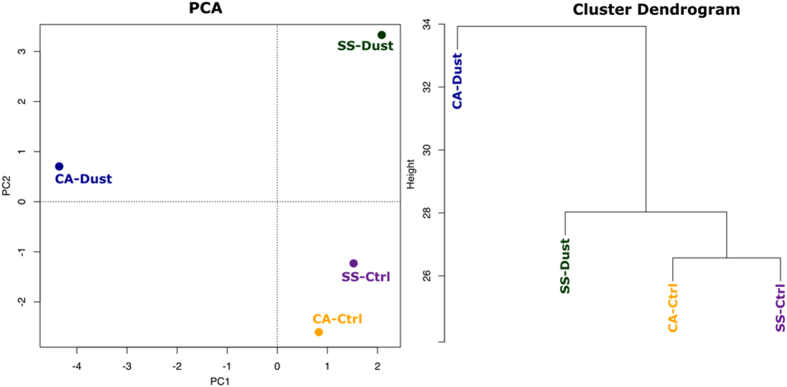
Principal Component Analysis (left) and Cluster Analysis based on euclidean distance, with average linkage method (right) based on the identified genera.

Principal Component Analysis (PCA) and cluster analysis (Fig. 4) define groups where the dust-related samplings occupy distinct positions while the two controls occur in a closer range of the ordination plot. The cluster analysis also shows shorter distances between the two controls and longer branches separating the dust samples, in particular for the one collected in the most southern location (Cagliari).

**Figure 5:**
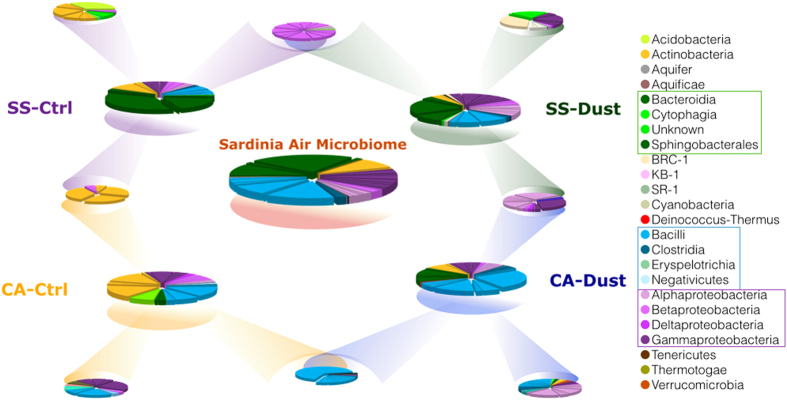
Proportions of identified phyla and classes in the four sampling cases. The legend on the right shows the color codes of the phyla, or, for three of them, the occurring classes listed within boxes in the legend as follows: Bacteroidetes (green tints); Firmicutes (blue tints); Proteobacteria (purple tints). The larger central pie chart shows the situation of the constant core of taxa shared by all four cases, constituting the putative Sardinia air microbiome, supposedly independent from place and weather conditions. The four inner-diamond small pies show the taxa shared pairwise by the ‘locality pairs’ (Cagliari or Sassari) or by the ‘whether condition pairs’ (dust or control). The four outer-corners small pies show the unshared (specific) fraction of each case. Pies diameters not drawn in scale with sequences frequency number.

The taxonomical composition of the core bacterial microbiome in comparison with the locality-shared, whether event-shared, or uniquely occurring groups at phylum and class level is shown in Fig. 5.

Concerning the identities of specific genera within the relevant phyla, as shown Actinobacteria were identified in all the conditions with lower percentages in the two dust-related samples, 7.5% and 3.8%, for Cagliari and Sassari respectively, and higher for the two controls, 14.8% and 11.0%. Within this major phylum the genus *Propionibacterium* represented the most abundant. This taxon was previously reported as desert dust particles^41^ and particles-associated pollutants but our data indicate higher percentages in the two controls. These two samples shared moreover an amount of other exclusive Actinobacteria genera.

On the other hand, interestingly, higher percentages of *Corynebacterium*, already detected in the Saharan air dispersed-particles^10^,^29^ characterized the two dust-related samples.

Members of the Bacteroidetes phylum were identified in all the datasets with percentages varying from 16% (dust) to 28% (control) in the two Cagliari samples while in the Sassari samples, these values were about double as Bacteroidetes-related reads represented from 32% to 59% of the total uniquely-aligned reads in the dust-related and in the control-sample respectively. Several authors observed their environmental abundance^42^. In terms of taxa variability it is worth noticing in the analyzed datasets, that although different at genus level within each sample the relative percentages of the Bacteroidetes in control vs. dust samplings, remain surprisingly unaltered.

About Firmicutes, the two Cagliari samples showed higher percentages, with a maximum that reaching the 57% for the dust-related samples and 30% for the negative control. Lower values were shown in Sassari, with percentages between 20% and 12% respectively. This phylum was often reported as one of the most abundant in the air-collected samples^7^,^41^,^43^. The *Bacillus* genus was detected but it does not represent the most abundant. High percentages of *Streptococcus* and *Lactococcus* characterized both Cagliari samples and were always detected in lower percentages; these genera had rarely been reported as particle associated in literature. Moreover, several Firmicutes like *Anoxybacillus* were identified as specific taxa characterizing the Cagliari samples.

A different situation was observed for the Proteobacteria phylum: an opposite trend was evident analyzing the four samples: percentages, from 16% to 28%, characterized the two Cagliari samples, dust and control, respectively, while opposite proportions, from 39% to 16% occurred the two dust-related and control from Sassari. Notwithstanding the phyla disproportions, genera distributions within them did not show evidence of consistent trends or correlations.

Interestingly, several groups were shared between the two dust-related samples. An amount of Alphaproteobacteria and Gammaproteobacteria characterized all the analyzed conditions but within this phylum several differences between dust-events and controls were detected. In particular, Alphaproteobacteria were found as dust-related samples. Taxa previously reported as desert-dust associated as *Paracoccus*^23^,^29^,^30^,^41^, *Sphingomonas*^10^,^23^,^41^,^44^, *Methylobacterium*^30^ or never detected before in these environments, as *Caulobacter* and *Brevundimonas* appeared to characterize the samples.

Some exceptions were also encountered: some Gammaproteobacteria like *Citrobacter*, *Cronobacter*, *Klebsiella*, *Pantoea* and *Stenotrophomonas* were shared between the Cagliari-control and the Sassari dust-related samples.

These shared genera flanked an amount of other Alpha and Gammaproteobacteria that chaacterize all the analyzed conditions, indicating a broad distribution and their relevance in the Sardinian air-microbiome definition.

## Discussion

As a first consideration, the analysis revealed that a wide group of phyla, recurrently consistent in spite of site and meteorology conditions, configures as a global Sardinian air microbiome. Exceptions were related to the intra-phylum biodiversity and the shared taxa between groups of samples.

Notwithstanding the persisting core, it was possible to observe that biodiversity increased in relation to dust-carrying events. As Table 2 shows, Shannon and Simpson richness indexes show upshifts in both localities in coincidence with the particle-carrying winds from the African side.

An enrichment in specific bacterial lineages could be determined by both weather factors and the local geographic distributions as exemplified by the percentages of Firmicutes characterizing the two Cagliari samples. On one hand, a background of bacteria already recognized as air-particles associated (e.g. *Propionibacterium*) could be representative of taxa that normally use the air-dispersion as mechanism of diffusion. On the other hand, the effects of a dust-event could determine an enrichment concerning specific bacterial populations like *Corynebacterium*, *Paracoccus* or *Brevundimonas*.

Biodiversity indexes confirmed that higher values characterized both the dust-related samples, Sassari and Cagliari (Table 2), underlining that an increasing biodiversity can be transported during a dust event, with a possible influence in modulating the composition of the local microbiome.

PCA and cluster analysis described a correlation between the two control samples while the dust-related taxa occupy different positions (Fig. 4).

About potential clinically-relevant bacteria we detected different cases of Gammaproteobacteria as *Citrobacter*^45^,^46^,^47^, *Cronobacter*^48^,^49^,^50^, *Klebsiella*, *Pantoea*^51^,^52^,^53^ and *Stenotrophomonas.* Within these, only *Pantoea* and *Stenotrophomonas*^54^,^55^,^56^ had been previously detected as air-particle associated^30^, but the others do represent warning-worth occurrences. The *Klebsiella* genus encompasses in this respect a group that can determine nosocomial infections because of its air-dispersion^57^,^58^.

The main points arising from the present study can be summarized as follows. A major conserved contingent of bacteria has been identified as representative of a local-air microbiome which appears to characterize at least the Sardinian pool of the atmospheric prokaryotes as it occurred in the two northernmost and southernmost sampling stations of the island irrespective of the wind direction and regime.

Nevertheless, dust outbreaks conveying air from Saharan Africa, do increase the local biodiversity. Such immigrations are testified by upshifts in the richness ecological indicators of the sampled air and by the detectable presence of specific taxa missing from the background resident microbiome.

The culture independent method of analysis has proven 1) sensitive in comparison with the performances attained by culture based plating methods, 2) suitable to reveal differences between localities and weather conditions, and 3) informative to achieve identities of the incoming species via bioinformatics annotation pipeline.

Some of the bacteria encountered do belong to clinically relevant taxa that can possess virulence properties and pathogenic behavior towards humans. The monitoring principle assumes therefore importance in programs of disease prevention against epidemiological outbreaks from affected regions and countries as well as a warning routine in the event of deliberate bacterial warfare and bio-terrorism attacks.

Further investigations are required to refine the composition and the boundaries of the conserved air microbiomes and to put in evidence their seasonal fluctuations.

## Materials and Methods

### Sampling sites

The Sardinia Island extends from 38.86° N to 41.31° N and 08.14° to 9.84° E.

Sampling sites were located in Cagliari (39.23°N, 9.11°E), located on the southern coast facing the African continent, and Sassari (40.75°N, 8.49°E) which represents, on the opposite side, the first outpost site reached by the north-western winds. The two sampling locations are 174 km apart and the distance covers essentially the whole longitudinal territorial length of the island.

### Meteorology forecasts

The predictive evaluation and alert of the Saharan dust discharge events was carried out by continuous monitoring of MODIS satellite data and Meteosat imagery combined with SKIRON^59^ forecasting model. SKIRON is a version of the ETA/NCEP weather forecasting model developed at the University of Athens with a forecast horizon of 5 days.

The origin and the back-trajectory plots of the dust carried by winds towards Italy were inferred by the NOAA HYSPLIT model (Hybrid Single Particle Lagrangian Integrated Trajectory Model)^60^,^61^. In addition, PM10 (particulate matter with a diameter of less than 10 μm) and meteorological data registered by the ARPAS (Regional Environmental Protection Agency of Sardinia) monitoring stations were used to highlight the arrival of African air masses.

Sampling was performed using a Skypost Tecora air-filtering apparatus; filters were collected in sterile boxes and stored at −20 °C until nucleic acids extraction.

### PCR and Sequencing

Filters processed for DNA analyses were selected based on the observed weather conditions and the backward trajectories analysis.

Two filters for each specific weather event were considered in each sampling site. Filters were collected in order to represent northbound dust outbreaks reaching Cagliari or Sassari (CA-Dust and SS-Dust, respectively), whereas the dust-negative controls were taken at each of the two above sampling stations under northwesterly wind (CA-Ctrl and SS-Ctrl). Two replicates per site and per condition were taken for a total of eight filters.

DNA was extracted using the E.Z.N.A.® Soil DNA Kit (Omega Bio-Tek Inc.) and following the manufacturer’s protocol. Quality and quantity of the extracted nucleic acids were measured using a NanoDrop 2000 spectrophotometer (Thermo Fisher Scientific Inc.).

Amplification of the 16S-rRNA genes was performed using the universal primers 27F-1492R (AGAGTTTGATYMTGGCTCAG and TACGGYTACCTTGTTACGACTT, respectively). PCR was performed using Platinum® Taq DNA Polymerase High Fidelity (Life Technologies) in a PTC-200 Thermal Cycler (MJ Research Inc.) set as follows: 95 °C for 5 min, (95 °C for 0.5 min, 51 °C for 0.5 min, 72 °C for 2 min for 30 cycles), 72 °C for 10 min and 4 °C hold.

Next generation sequencing was done at the Porto Conte Ricerche Srl (Alghero, Italy). Briefly, amplicons were quality-checked on an agarose gel and purified using the Agencourt® Ampure® XP PCR Purification Kit. One ng of DNA was processed using the Nextera XT DNA Sample Preparation Kit (Illumina Inc.) and sequenced using the HiScanSQ (Illumina Inc.) with 93 bp × 2 paired-end reads.

### Data Analysis

A bioinformatic pipeline based on a two-step alignment-and-selection strategy was developed in order to classify and quantify each bacterial group.

Illumina reads were quality filtered and aligned against the SILVA reference database^62^ by using BWA^63^.

Sequences covered at least for the 10% of the total length by uniquely-aligned reads were firstly selected, obtaining a new dataset of putative subjects. Using these as a reference database, a second BWA alignment was performed and sequences covered at least for the 30% of the total length were then considered to give the final result. The naive 16S-rRNA genes classification attributed by the SILVA database was applied for the subjects taxonomic assignment. The percentage of uniquely-aligned reads on the total number of uniquely-aligned reads for each sample was considered to estimate and quantify each taxa.

## Culture-dependent analyses

In order to characterize in parallel the fraction of culturable biota, some of the filters were placed, sample-side up, cut and cleaned with Tween 20. The filters pieces were removed and placed in selective and non-selective agar and incubated at room temperature for 24–72 hours for bacteria and 15–30 days for fungi. The media used were: Blood Agar, Chocolate Agar, Mannitol salt Agar, Mac Conkey agar, Thioglycollate medium usp with resazurine for bacteria and Sabouraud dextrose agar + caf + gent for fungi (Microbiol Diagnostici, Macchiareddu Uta Italy).

Cultured taxa were identified starting from a single colony using Matrix-Assisted Laser Desorption Ionization Time of Flight Mass Spectrometry (MALDI TOF, Microflex LT Bruker), Vitek2 system and optical microscopy for colony morphology assessment.

The MALDI TOF technology is an alternative to classical microbiological identification for bacterial isolates, whose recognition is accomplished through comparison of the peak lists with a library containing the spectra information of species. It has been successfully used for species borne in air samples from Arabia^64^. Nevertheless it is recommended to couple its use with complementary techniques as the MALDI-TOF database is still incomplete as regards spectra of a number of species, e.g. some of the non-pathogenic Bacillus species. Therefore the Vitek2, providing automated identification based on antibiotic susceptibility profiles, was used as a suitable companion technique which can provide targeted phenotypic tests, in particular those covering aerobic endospore-forming bacteria^65^.

## Additional Information

**How to cite this article**: Rosselli, R. *et al.* Microbial immigration across the Mediterranean via airborne dust. *Sci. Rep.* **5**, 16306; doi: 10.1038/srep16306 (2015).

## Supplementary Material

Supplementary Information

Supplementary Table S1

## Figures and Tables

**Table 1:** Total number of sequences and uniquely aligned reads obtained.

Sample	CA-Dust	SS-Dust	CA-Ctrl	SS-Ctrl
n. of paired reads	1’331’182	1’664’672	1’270’770	1’125’836
n. of uniquely aligned reads	756’655	1’051’021	780’713	644’623
PRJNA274749 (Bioproject)	SAMN03332112	SAMN03332148	SAMN03332147	SAMN03332149

**Table 2:** Number of genera detected in the four cases and species diversity indexes.

Samples	CA-Dust	CA-Ctrl	SS-Dust	SS-Ctrl
N. of Genera	345	232	293	259
Simpson Index	0.891	0.910	0.931	0.818
Shannon Index	3.204	3.086	3.487	2.717
