# Supplementary material for: Microbial immigration across the Mediterranean via airborne dust
Source: Sci Rep. 2015 Nov 6;5:16306. doi: 10.1038/srep16306 (PMC4635359; doi:10.1038/srep16306)
Supplement: Supplementary Information [file srep16306-s1.pdf]

SUPPORTING INFORMATION

Microbial immigration across the Mediterranean via airborne dust

Riccardo Rosselli[1], Maura Fiamma[2], Massimo Deligios[2], Gabriella Pintus[3], Grazia Pellizzaro[3] Annalisa Canu[3], Pierpaolo Duce[3], Andrea Squartini[5*], Rosella Muresu[4], Pietro Cappuccinelli[2]

[1]Department of Biology, University of Padova, Via Ugo Bassi 58/b, 35131 Padova, Italy [2]Department of Biomedical Sciences-University of Sassari, Italy, [3]Institute of Biometeorology-National Research Council (IBIMET-CNR), Italy, [4]Institute of Animal Production Systems in Mediterranean Environments-National Research Council (ISPAAM-CNR),Italy. [5]Department of Agronomy Animals, Food, Natural Resources and Environment, DAFNAE, University of Padova, Viale dell'Università 16, 35020 Legnaro (Padova) Italy *Corresponding author: squart@unipd.it

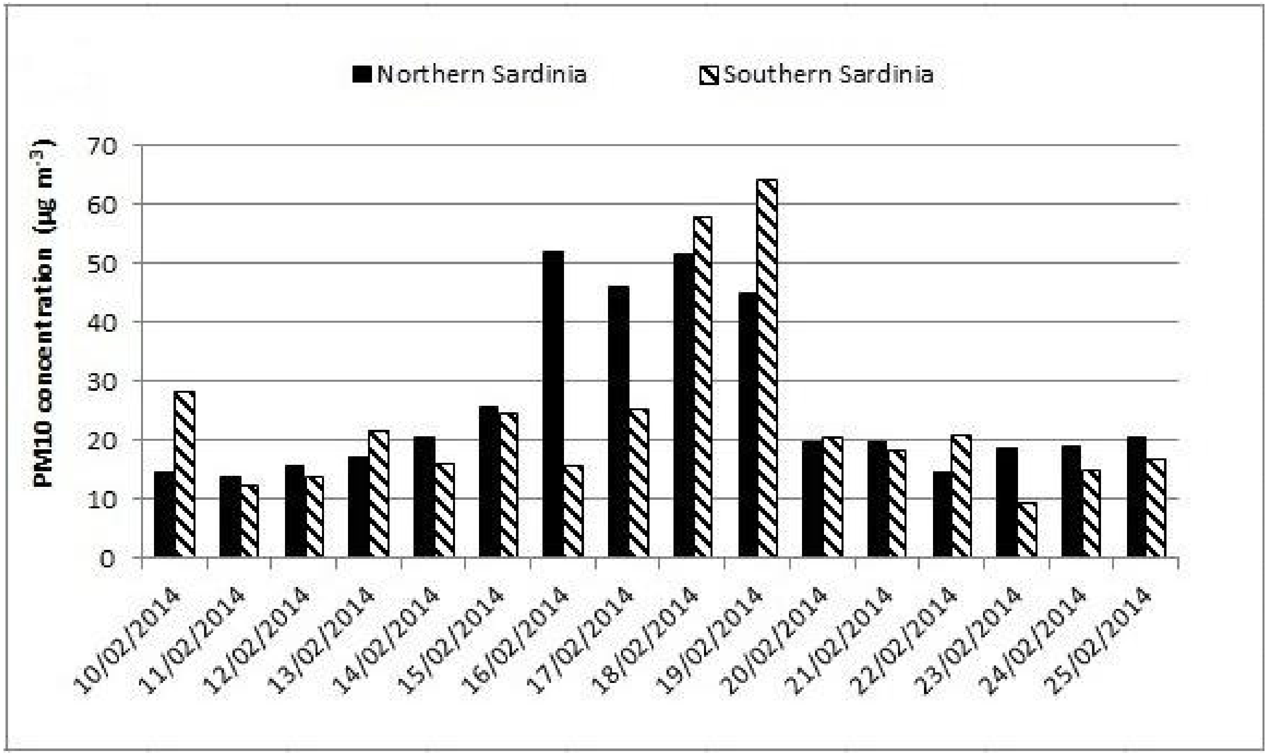

Figure S1 Daily PM_{10} values recorded during the period 10-25 February 2014 at the monitoring stations located in Northern Sardinia (40.72°N, 8.55°E) and Southern Sardinia (39.23°N, 9.00°E).

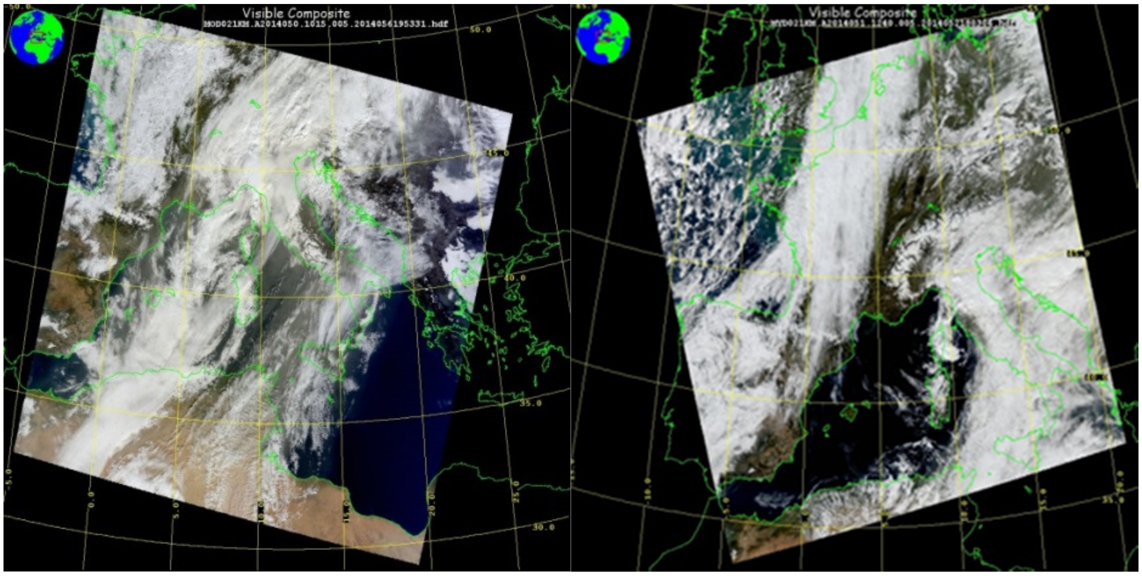

Figure S2. Satellite images (MODIS), 19th and 20th February 2014 (credit to: http://modis.gsfc.nasa.gov/data/)
